# Highly Sensitive Multi-Channel IDC Sensor Array for Low Concentration Taste Detection

**DOI:** 10.3390/s150613201

**Published:** 2015-06-05

**Authors:** Md. Rajibur Rahaman Khan, Shin-Won Kang

**Affiliations:** School of Electronics Engineering, Kyungpook National University, 1370 Sankyuk-Dong, Bukgu, Daegu 702-701, Korea; E-Mail: rajibur@ee.knu.ac.kr

**Keywords:** interdigitated capacitor, dielectric constant, taste sensor array, principal component analysis

## Abstract

In this study, we designed and developed an interdigitated capacitor (IDC)-based taste sensor array to detect different taste substances. The designed taste sensing array has four IDC sensing elements. The four IDC taste sensing elements of the array are fabricated by incorporating four different types of lipids into the polymer, dioctyl phenylphosphonate (DOPP) and tetrahydrofuran (THF) to make the respective dielectric materials that are individually placed onto an interdigitated electrode (IDE) via spin coating. When the dielectric material of an IDC sensing element comes into contact with a taste substance, its dielectric properties change with the capacitance of the IDC sensing element; this, in turn, changes the voltage across the IDC, as well as the output voltage of each channel of the system. In order to assess the effectiveness of the sensing system, four taste substances, namely sourness (HCl), saltiness (NaCl), sweetness (glucose) and bitterness (quinine-HCl), were tested. The IDC taste sensor array had rapid response and recovery times of about 12.9 s and 13.39 s, respectively, with highly stable response properties. The response property of the proposed IDC taste sensor array was linear, and its correlation coefficient *R*^2^ was about 0.9958 over the dynamic range of the taste sensor array as the taste substance concentration was varied from 1 μM to 1 M. The proposed IDC taste sensor array has several other advantages, such as real-time monitoring capabilities, high sensitivity 45.78 mV/decade, good reproducibility with a standard deviation of about 0.029 and compactness, and the circuitry is based on readily available and inexpensive electronic components. The proposed IDC taste sensor array was compared with the potentiometric taste sensor with respect to sensitivity, dynamic range width, linearity and response time. We found that the proposed IDC sensor array has better performance. Finally, principal component analysis (PCA) was applied to discriminate different types of taste of the mixed taste substances.

## 1. Introduction

An electronic taste sensor is a device with an array of systems that are able to detect single taste substances, as well as complex mixtures of substances. Such multichannel taste sensors, which are referred to as electronic tongues, taste sensing systems, electronic taste sensor array systems, taste chips or biomimetic sensor array systems, are believed to determine taste in a manner similar to biological taste perception in humans [1,2].

Electronic tongue/taste sensors have been used by the food and beverage industry, pharmaceutical industries and research institutes and by developers of environmental monitoring, medical diagnostics and safety applications to develop and manage the quality of new products. In the food industry [3,4,5,6,7,8], electronic taste sensors are used for quality control and in the comparison of the quality of different products, including those produced by competitors. In diagnostic centers, taste sensors are used to measure the sugar in blood and urine, and in environmental monitoring [9,10,11] centers and research institutes, taste sensors are used to observe water quality parameters, including: the presence of inorganic, organic and radioactive pollutants; chemical and metabolic breakdown patterns; the presence of harmful bacteria and substances; and potential origin information on pesticides, oil, dioxins, *etc.* Taste sensors are also used in waste monitoring and petrochemical processing [12].

More technically, an electronic tongue can be defined as an instrument, comprised of an electrochemical cell, a sensor array and an appropriate pattern recognition system that is capable of recognizing the simple or complex soluble nonvolatile molecules that form the taste of a sample [13]. Electronic tongue systems can be based on various measuring principles, including potentiometry [14,15,16], voltammetry [17,18,19,20] and amperometry [21]. To date, several studies have been performed using electronic tongues. For example, in 2009, Thete *et al.* proposed a fluorometric micro spot array, also called an optochemical tongue, for recognizing different alcoholic beverages [22]. Their taste micro spot was composed of binary mixtures of different fluorescence dyes that were embedded in a hydrogel matrix. Although the array is simple and inexpensive to fabricate, it is bulky, and it uses a complex detection process. A capillary-based microbead electronic tongue [23] was introduced by Sohna *et al.* The advantages of this electronic tongue include its small size, low cost and real-time monitoring ability; however, the electronic tongue also has several disadvantages, including a complex fabrication process and the need for a light source and detector. In 2002, Riul *et al.* proposed an impedance spectroscopy-based electronic tongue [24]. Although this tongue has high sensitivity, does not require a reference electrode and uses no electroactive materials in its sensing units, its sensing element relies on a complex sensing apparatus in which impedance is measured by varying the frequencies of a signal applied to an electrode sensor with an ultra-thin sensing membrane composed of several materials. Sehra *et al.* proposed a dual-shear horizontal surface acoustic wave (SH-SAW) [25] electronic tongue to discriminate among liquids having the different basic tastes of sour, salt, bitter and sweet. Although fabrication and operation of this electronic tongue is simple, its sensor has no selectivity. In 1998, Takagi *et al.* proposed a multichannel taste sensor whose transducer is composed of several kinds of lipid/polymer membranes [26]; while it can detect different tastes in a manner similar to human gustatory sensation, the sensor is very expensive and bulky.

There are two commercially available electronic tongue systems on the market: the SA402B [27] (Insent Inc., Atsugi-chi, Japan) taste sensing system, which is equipped with lipid membrane sensors; and the ASTREE [28] electronic tongue (Alpha M.O.S., Toulouse, France), which is based on chemical field effect transistor technology. Both of those sensing systems measure changes in the electronic potential of liquid samples.

Since the early 1970s, the technological application of interdigital capacitors/electrodes has received much attention in the literature, with the use of interdigitated capacitor (IDC) structures having accounted for a huge field of research on applications, including: dielectric studies on thin films [29]; microwave integrated circuits [30,31]; optically-controlled microwave devices [32]; optical and surface acoustic wave devices [33]; and tunable devices [34]. Recently, studies have been made on the use of IDCs as humidity [35] and chemical sensors [36,37,38,39,40]. Huang *et al.* proposed an interdigitated microelectrode (IDμE)-based microfluidic channel to detect different concentrations of glucose on a microelectrode surface without the use of immobilizing enzymes. Although its operating principle and construction are simple, the proposed sensor has no selectivity [41].

In our experiment, we designed a high-selectivity, low-cost and high-sensitivity IDC-based sensor array to detect various types of taste. The taste sensor array has four IDC sensing elements and uses an operational method based on the capacitor principle. Using four different types of lipid, such as oleic acid (OA), dioctyl phosphate (DOP), trioctylmethylammonium chloride (TOMA) and oleyl amine (OAm), incorporated into polyvinylchloride (PVC), dioctyl phenylphosphonate (DOPP) and tetrahydrofuran (THF), we fabricated the four dielectric materials of the IDC. These dielectric materials were then deposited individually via spin coating onto an interdigitated electrode (IDE) in order to produce the four IDC taste sensing elements of an array. In our experiments, dipping the array into a taste solution caused the dielectric material of the IDC to come into contact with the taste substance; this caused its dielectric properties to change as a result of a capacitance change in the IDC sensing element, which, in turn, changed the voltage across the IDC, as well as the output terminal voltage of each channel of the sensing system. The data from each channel were collected by a computer via a multifunction data acquisition (DAQ) module. We wrote a LabVIEW program to observe the sensing performance of the taste sensing system and to store data in the computer. Finally, we applied principal component analysis (PCA) [42,43] to distinguish different types of taste substance, with the results showing that good classification success was achieved for the various tested taste types. Our data confirmed the validity of our proposed sensor, which has the additional advantages of low fabrication cost, real-time monitoring capability and a linear sensing response over a dynamic range, while being compact and using electronic circuitry components that are readily available on the local electronics component market.

## 2. Theory and Operation Principle

**Figure 1:**
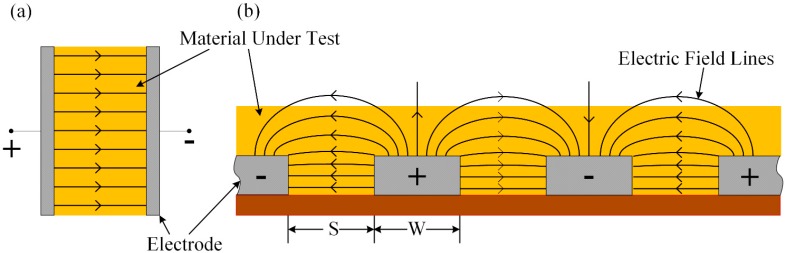
Electric fields of the (**a**) parallel plate capacitor and (**b**) coplanar interdigital sensor.

A schematic diagram of an IDE with a sensing membrane is shown in Figure 1. Placing the sensing membrane onto the IDE forms a capacitor, and the principle of operation of an interdigitated sensor is based on the electrodynamics of two parallel plate capacitors. To generate an electric field between the electrodes, an AC voltage source is applied between the positive and negative terminals. The generated electric field penetrates the material under test (MUT), which, in turn, changes the impedance of the sensor. Figure 1a,b shows the electric field configurations of a two parallel plate capacitor and an interdigitated sensor, respectively. Because the sensor behaves like a capacitor, its capacitive reactance is a function of the MUT; therefore, when a taste substance reacts with the sensing membrane (*i.e.*, the dielectric material) of the IDC, the IDC’s dielectric constant changes, which, in turn, changes the capacitive reactance of the sensor. Thus, by measuring the change in capacitive reactance or the voltage across the IDE, sensing behaviors can be observed. 

**Figure 2:**
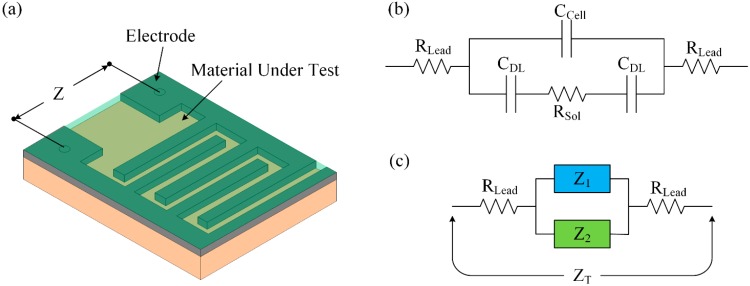
Interdigitated electrode: (**a**) configuration of the interdigitated impedance cell; (**b**) simplified electrical circuit of the interdigitated impedance cell; and (**c**) analogy of Figure 2b.

Figure 2 shows the configuration of the planar structure of an interdigitated impedance cell. A representation of the simplified equivalent electrical circuit of this impedance cell when it is immersed in an electrolyte is shown in Figure 2b. An analogy of the equivalent circuit in Figure 2b is shown in Figure 2c, which shows a circuit consisting of two double-layered capacitors (C_DL_) connected in series with a resistor of medium solution (R_Sol_), which, in turn, is connected in parallel with a dielectric capacitor (C_Cell_). The lead resistance R_Lead_ is the sum of the series resistances of the connecting wires. 

The resistance R_Sol_ of the medium serves as the sensing element and is a function of the electrolyte conductivity σ_Sol_ and the cell constant K_Cell_, as represented by the following equation [44]:

(1) $$R_{\mathrm{Sol}}=\frac{K_{\mathrm{Cell}}}{\sigma_{\text{Sol}}}$$

The cell constant K_Cell_ can be written using the following equation [45]:

(2) $${\text{K}}_{\text{Cell}}\text{=}\frac{2}{\text{(N-1)L}}.\frac{\text{K(k)}}{\text{K}\left(\sqrt{{\text{1-k}}^{2}}\right)}$$

where N and L are the number and length of fingers, respectively. The parameter K(k) is a complete elliptic integral of the first kind of the modulus k, which is defined respectively by:

(3) $$\text{K(k)=}\int_{0}^{1}\frac{1}{\left(\sqrt{\left({\text{1-t}}^{2}\right)\left({\text{1-k}}^{2}{\text{t}}^{2}\right)}\right)}\text{dt}$$

and:

(4) $$\text{k}=\cos\left(\frac{\text{π}}{2}.\frac{W}{S+W}\right)$$

or:

(5) $$\text{k}=\cos\left(\frac{\text{π}W}{\lambda }\right)$$

where S and W are the interelectrode space and width of the electrode, respectively. λ is a special wavelength defined as [46,47]:

(6) λ=2(W+G)

The direct capacitive coupling between the two electrodes is represented by the cell capacitance C_Cell_ and is represented in the following form [48]:

(7) $$C_{\mathrm{Cell}}=\frac{{\text{ε}}_{0}{\text{ε}}_{\text{r-sol}}}{K_{\mathrm{Cell}}}$$

where ${\text{ε}}_{0}$ and ${\text{ε}}_{\text{r-sol}}$ are the absolute and relative dielectric constants of the solution/medium, respectively.

The total impedance can be calculated from Figure 2c and expressed as:

(8) $$Z_{T}=2R_{\mathrm{Lead}}+Z_{P}$$

where:

(9) $$Z_{P}=\frac{Z_{2}}{1+\frac{Z_{1}}{Z_{2}}}=\frac{Z_{2}}{1+2\text{πf}C_{\mathrm{Cell}}Z_{2}}$$

Based on the electrical circuit in Figure 2c, the impedances Z_1_ and Z_2_ can be written as:

(10) $$Z_{1}=\frac{1}{2\text{πf}C_{\mathrm{Cell}}}$$

and:

(11) $${\text{Z}}_{2}=\frac{1+\text{πf}R_{\mathrm{Sol}}C_{\mathrm{Cell}}}{\text{πf}C_{\mathrm{Cell}}}$$

where f is the frequency of signal. At low frequencies, $Z_{P}\approx Z_{2}$; therefore, from Equation (9), we get,

(12) $$Z_{P}\approx \frac{1+\text{πf}R_{\mathrm{Sol}}C_{\mathrm{Cell}}}{\text{πf}C_{\mathrm{Cell}}}$$

At the high frequencies, $Z_{2}\approx R_{\mathrm{Sol}}$, in which case Equation (9) can be represented as:

(13) $$Z_{P}=\frac{R_{\mathrm{Sol}}}{1+2\text{πf}C_{\mathrm{Cell}}R_{\mathrm{Sol}}}$$

The voltage across the IDC sensor can be written using the following equation:

(14) $$V_{C}=I_{C}Z_{P}$$

where I_C_ is the constant current flow through the IDC sensor. The change in voltage across the IDC can be represented in the following form:

(15) $$\Delta V_{C}=I_{C}\Delta Z_{P}$$

## 3. Experimental Details

### 3.1. Fabrication of the Interdigitated Electrode

**Figure 3:**
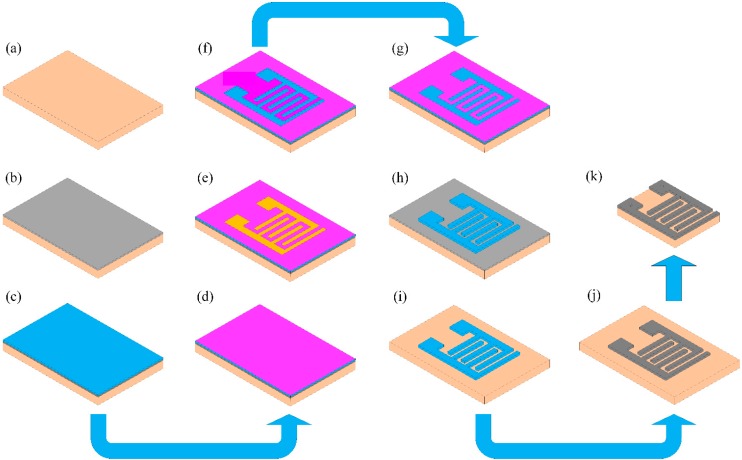
Step-by-step fabrication process of the interdigitated electrode: (**a**) polyimide substrate; (**b**) Cr layer on the polyimide substrate; (**c**) Cu layer; (**d**) photoresist layer; (**e**) placing the mask pattern on the photoresist layer; (**f**) transferring the mask pattern onto the photoresist layer; (**g**) depositing Cu via electroplating; (**h**) removing the photoresist; (**i**) removing the Cr layer; (**j**) depositing the Sn layer onto the Cu layer via electroplating; and (**k**) cutting the residual polyimide substrate.

**Figure 4:**
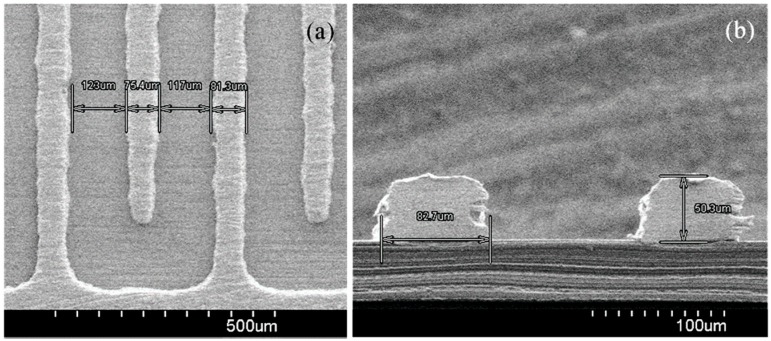
SEM images of the fabricated interdigitated electrode (IDE): (**a**) top view and (**b**) cross-sectional view of the IDE.

We fabricated the interdigitated electrode (IDE) used in our experiment on a polyimide (PI) substrate. Polyimide, e.g., Kapton^®^, is a high-performance polymer that has a number of desirable properties, including a high degree of thermal stability, chemical stability, low dissipation factor and good dielectric properties; as a result, polyimide can be used as substrate material for flexible printed boards, multilayer PCBs and ribbon cables. In our experiment, we used the Kapton^®^ HN-type polyimide substrate with a thickness of 5 mil, because it exhibits an excellent balance of physical, chemical and electrical properties over a wide temperature range. The fabrication process of the IDE on a PI substrate is described step-by-step below and represented schematically in Figure 3. The PI substrate was first cleaned with ethanol, methanol and deionized (DI) water. Then, a thin (15 nm) layer of chromium (Cr) was grown on the PI substrate via a vacuum evaporation process (the advantage of using Cr is that it provides superior adhesion performance). Next, we deposited an approximately 100 nm-thick copper layer onto the Cr layer; in turn, the Cu-layer was covered with photoresist SU-8 25 or AZ-4620 using a spin coater, with the choice of photoresist based on which was suitable for molding a relatively high aspect ratio electrode structure. Then, a Karl Suss MJB3 UV300 mask aligner was used to transfer a mask pattern onto the Cu-layer via UV exposure. Using a copper electroplating solution at an applied current density of 45 mA/cm^2^, a copper (Cu) layer was electroplated onto the Cu layer, after which the photoresist mold, unnecessary Cu layer and the bottom layer of Cr were removed using solvent suitable for avoiding short-circuiting. Finally, the Cu-interdigitated electrode was overlapped by a very thin layer of electroplated tin using an electroplating solution. In our experiment to deposit the Cu layer properly on the Cr layer, firstly, we deposited a very thin of the Cu layer on Cr layer, then we electroplated a thick layer of Cu of approximately 50 µm on the thin Cu layer. This step also reduces the fabrication cost and time. Tin is a useful metal that has non-toxic, ductile and corrosion resistance properties. Tin also has the ability to protect the Cu from oxidation. Thus, in our experiment, we deposit a thin layer of tin of approximately 30 nm on Cu IDE. Since the capacitance of a capacitor depends on the gap between the two plates, in our experiment, firstly, we selected/fixed the width W of the fingers to be about 80 µm. Then, we changed the space S between the electrodes with different values, such as S = W = 80 µm, S = 0.5 W, S = 1.5 W and S = 2 W, to observe the performance of the IDC and found that when S = 1.5 W = 120 µm, then the IDC offers better performance. That is why, in our study, we selected the gap between the fingers to be about 120 µm. The thickness of the fabricated IDE was approximately 50 μm, with gaps between fingers of about 120 μm and a width per finger of approximately 80 μm, as measured by a scanning electron microscope (S-4800, Hitachi, Ibaraki, Japan). SEM images of the top view and cross-sectional view of the fabricated IDE is shown in Figure 4a,b, respectively.

### 3.2. Fabrication of the Sensing Solution and Interdigitated Sensing Element of the Array

**Figure 5:**
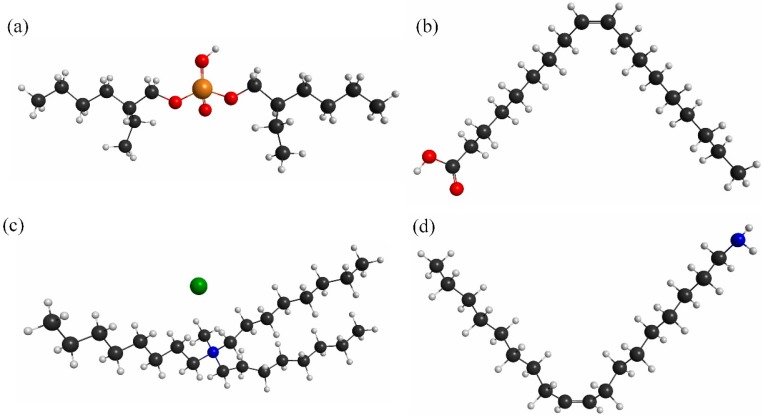
Molecular structures of the lipids: (**a**) oleic acid; (**b**) dioctyl phosphate; (**c**) trioctylmethylammonium chloride; and (**d**) oleyl amine.

In our experiment, four types of lipids, namely oleic acid (OA), dioctyl phosphate (DOP), trioctylmethylammonium chloride (TOMA) and oleyl amine (OAm), and one polymer from polyvinylchloride (PVC), dioctyl phenylphosphonate (DOPP) and tetrahydrofuran (THF) were used to prepare the dielectric materials/sensing solutions for the four sensing elements of the array. In our experiment, when the lipid reacts with a taste substance, then its chemical properties change; as a result, the dielectric properties of the solution, as well as the dielectric properties of sensing membrane change, which also changes the capacitance of the IDC taste sensing element. The used lipids are popular and give better performance than other lipids, which is why we chose those four lipids. We purchased all reagents from the Sigma-Aldrich Chemical Corporation and used them without further purification. Table 1 lists the compositions of the sensing solutions for the four IDC taste sensing elements of the sensor array. The molecular structures of the lipids are shown in Figure 5. For the sensor elements (S1 to S4), we prepared four types of sensing solution using the following procedure. First, we dissolved 500 mg of PVC into 10 mL of THF and sonicated for about 10 min to make Solution A. Then, we added 0.5 mL of DOPP to Solution A and sonicated again for about 10 min to prepare Solution B. After that, we took 2.5 mL of Solution B and added 0.6 µL of OA and sonicated about 15 min to make the sensing solution for IDC sensing element S1. Similarly, we added 0.6 µL of DOP, 0.56 µL of TOMA and 0.6 µL of OAm individually into 2.5 mL of Solution B to make the sensing solution or dielectric solution for the S2, S3 and S3 sensing elements of the array, respectively. The IDE was washed with acetone, methanol and DI water and dried with N_2_ gas. We used a spin-coater to place the sensing solution into the IDE over three stages of spinning, 500 rpm for 5 s, 1000 rpm for 5 s and, finally, 1500 rpm for 20 s, to fabricate the IDC. After deposition of the sensing solution, the IDC was dried overnight at room temperature. 

**Table 1:** The composition of each sensing solution of the sensor array. OA, oleic acid; DOP, dioctyl phosphate; TOMA, trioctylmethylammonium chloride; OAm, oleyl amine; PVC, polyvinylchloride; DOPP, dioctyl phenylphosphonate; THF, tetrahydrofuran.

Sensor ID	Lipid	Polymer	Solvent
S0		PVC	DOPP and THF
S1	OA	PVC	DOPP and THF
S2	DOP	PVC	DOPP and THF
S3	TOMA	PVC	DOPP and THF
S4	OAm	PVC	DOPP and THF

### 3.3. Detection System

**Figure 6:**
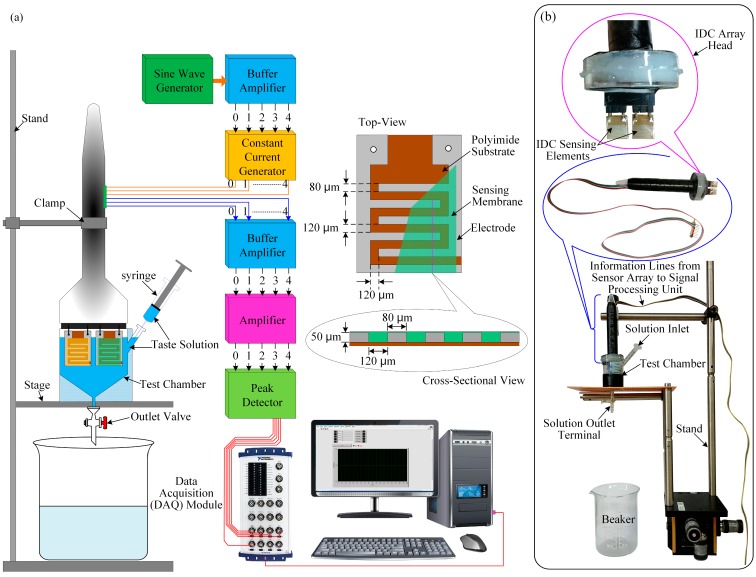
Experimental setup of the IDC array taste sensing system: (**a**) schematic diagram of the taste sensing system; and (**b**) photograph of the different parts of the IDC sensor array.

A schematic diagram of the proposed interdigitated taste sensing system for the characterization of different taste substances is shown in Figure 6. It consists of a test chamber, four interdigitated taste sensing elements, one reference IDC element, a signal processing unit, an oscilloscope (TDS3032B, Tektronix, Wilsonville, OR, USA), a multifunction data acquisition (DAQ) module (NI USB-6216 BNC, National Instruments, Debrecen, Hungary) and a PC. The signal processing unit is primarily divided into five parts: an oscillator, buffer amplifier, constant current generator, amplifier, and peak detector [49,50,51]. We designed all electronic circuits of the taste sensing system to use readily available and inexpensive electronic components.

The sensor array consists of four interdigitated taste sensing elements containing the lipid mixed-polymer sensing membrane. The voltage (ΔV) measured over each of the four sensors is the difference between the voltage of the taste sensing element and that of the reference signal voltage. 

For our experiment, we designed a sine wave generator with a frequency of 1 kHz. The output of the sine wave generator is connected to the input of a buffer amplifier, which has high input impedance and low output impedance in order to remove/reduce the loading effect. The buffer amplifier output is fed to the inputs of the five constant current sources, each of which is connected to a different taste sensing element and reference element. The voltage across each IDC taste sensing element is applied to the input of the buffer amplifier, the output of which is fed to the amplifier to obtain sufficient voltage amplification. The amplified signal is then applied to the input of the peak detector for conversion to DC voltage. The signal processing unit is connected to the PC via the DAQ module.

Four different types of taste substances, namely HCl, NaCl, glucose and quinine-HCl, are mixed with aqueous solutions to obtain the desired concentrations of the target taste solution from 1 µM to 1 M. Most of the components of the test chamber are composed of Teflon, which is used to prevent the target taste solutions from being absorbed by the container. The taste sensor array, also called the electronic tongue, is held in vertically within test chamber/container by a clamp. A syringe is used to inject the taste substance into the test chamber, while an outlet valve is used to remove the taste solution after measurement or cleaning of the test chamber and the electronic tongue; during measurement, the outlet valve is closed. To test the response of the sensor array/electronic tongue, we inject a reference solution via syringe into the test taste chamber to obtain a stable baseline, following which, a target taste solution is injected slowly into the chamber to obtain the response baseline. Upon exposure to the target taste solution, the sensing membranes (dielectric material) of the sensor array come into contact with the taste solution, causing the dielectric constant, and, thus, the capacitance, of the IDC sensing membrane to change depending on the concentration of the taste solution. The change in capacitance of the IDC causes the voltage across the IDC to change, which, in turn, changes the output voltage of the received sensing signal. The five outputs of the peak detectors are connected to inputs of the DAQ module connecting to the PC; a LabVIEW program is used to observe the real-time response of the taste sensor array and to record the results.

## 4. Results and Discussion

**Figure 7:**
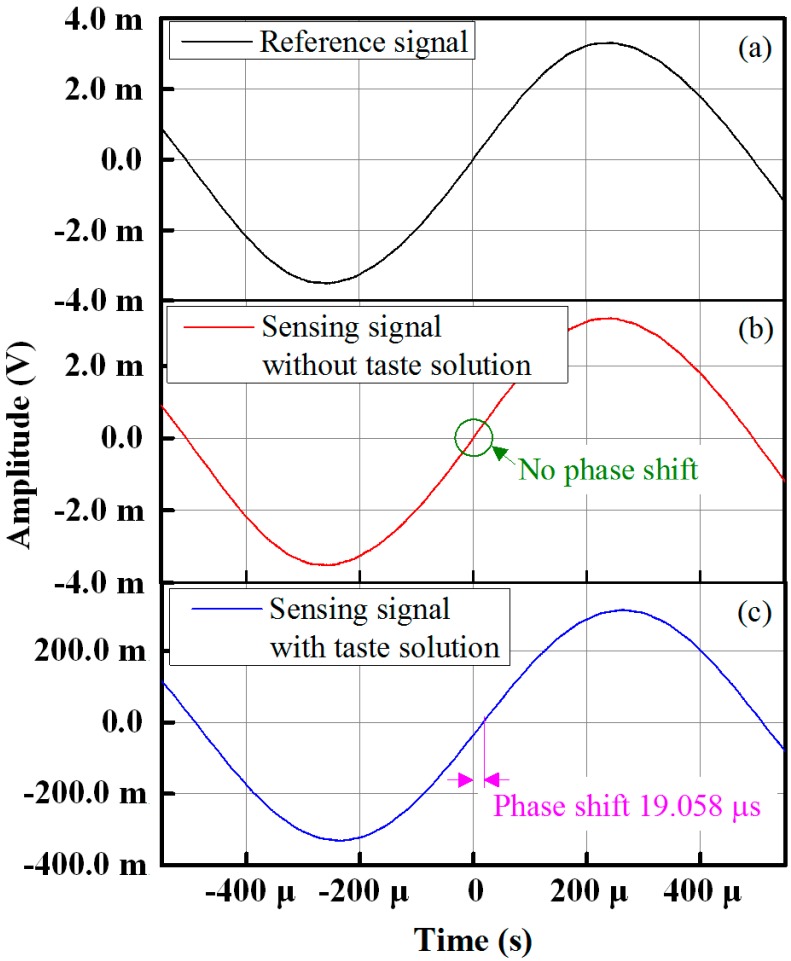
Waveform response of sensing and the reference signals: (**a**) reference signal; (**b**) response before injecting taste solution; and (**b**) response with taste solution.

From Figure 7a,b, which shows the sensing and reference signal waveforms under ideal conditions (*i.e.*, with no taste solution in the test chamber), it is seen that there is no phase difference between the sensing and the reference signals and that the amplitudes are almost identical. From Figure 7c, injecting a taste sensing solution into the taste chamber causes a phase shift to occur between the sensing signal and the reference signal, as measured using the oscilloscope (Tektronix TDS3032B). From the figure, it is seen that the phase shift difference between the reference signal and a sensing signal with an HCl concentration of 100 mM is 19.058 µs. The waveform results in Figure 7 indicate that the performance of the signal processing unit of the proposed IDC taste sensing system is good and that the sensor is capable of detecting small differences in both the phase shift and amplitude of a received sensing signal.

**Figure 8:**
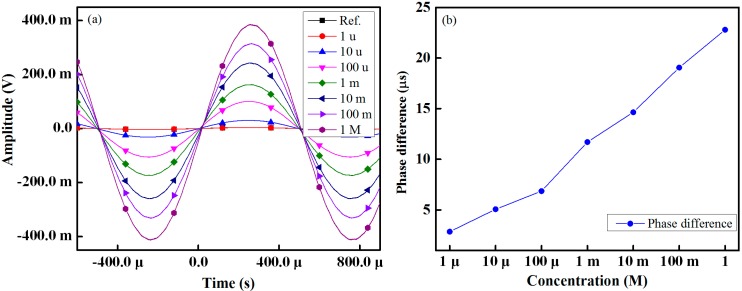
Performance of proposed IDC taste sensing: (**a**) amplitude across the IDC taste sensing element at different concentrations of HCl; and (**b**) change in the phase shift.

From Figure 8a, it is seen that increasing the HCl concentration also increases the amplitude of the signal across the IDC. Variations in the phase shift of the sensing signal with respect to the reference signal at HCl concentrations ranging from 1 µM to 1 M are shown in Figure 8b, from which it is readily apparent that, as the concentration of HCl increases, the phase shift between the two signals increases in a linear manner. 

We were able to determine the electrical and optical characteristics of the sensing solutions, and we found that increasing the amount of PVC in the sensing solution turns out to reduce the conductivity of the solutions and *vice versa*. The electrical property, *i.e.*, conductivity of the used OA, DOP, TOMA and OAm lipid-containing sensing solution was 0.039, 0.058, 1.060 and 0.019 mS/m, respectively. Similarly, the optical property, *i.e.*, refractive index of the used OA, DOP, TOMA and OAm lipid-containing sensing solution was 1.154, 1.98, 5.76 and 0.9169, respectively. To observe the reproducibility performance of the sensing membrane, we prepared three samples of the sensing solution, which contained OA lipid, and after that, we placed those solutions into three IDEs to make three IDC taste sensing elements. Then, we observed the electrical properties, as well as sensing performance of the three OA containing IDC, and we found that the three OA-containing IDC sensing elements show almost the same sensing performance. Therefore, it can be concluded that the sensing membrane has good reproducibility. Table 2 shows the statistical data of the proposed IDC taste sensor array of the three observations of three samples of OA-containing sensing membranes.

**Table 2:** Statistical data of the proposed IDC taste sensor array of the three observations of three samples of OA-containing sensing membranes.

Observation No.	Sample’s Name	Dielectric Constant	Capacitance (pf)	Standard Deviation of the Dielectric Constant
1	OA_1_	1.33290	1.18015	
2	OA_2_	1.38996	1.24105	0.029
3	OA_3_	1.35165	1.18996	

**Figure 9:**
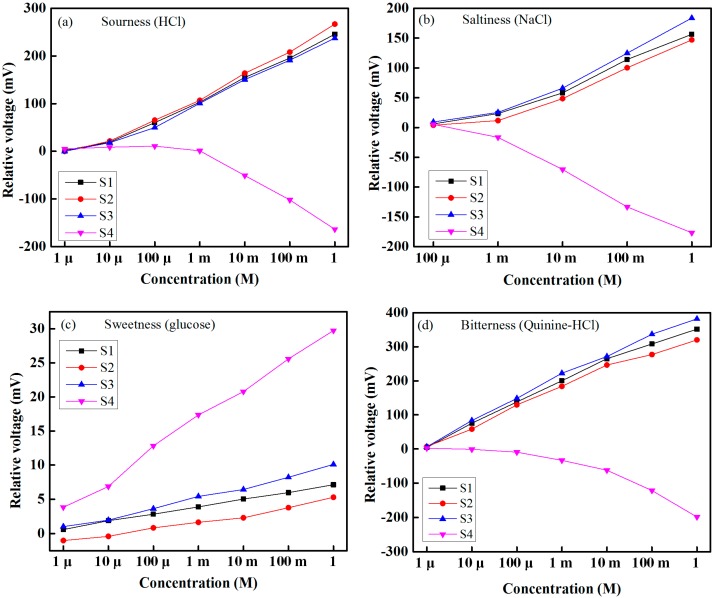
Responses of the sensing elements of the array with respect to type of taste and degree of concentration: (**a**) sourness (HCl); (**b**) saltiness (NaCl); (**c**) sweetness (glucose); and (**d**) bitterness (quinine-HCl).

To observe the performance of each individual sensing element of the sensor array with respect to taste solution, we injected different solutions individually and at varying concentrations into the taste chamber. All taste solutions were detected at room temperature. Figure 9 shows the response of the four sensors at taste solution concentrations of 1 µM to 1 M for sourness (HCl), sweetness (glucose) and bitterness (quinine-HCl) and taste solution concentrations of 100 µM to 1 M for saltiness (NaCl). The response of a sensor is the difference between the obtained voltage of the sensor and that of the reference sensor. From Figure 9, it is seen that the relative sensing voltage increases as the concentration of taste solution increases, and *vice versa*. It can be determined from these experimental results that each sensing element of the array offers a linear sensing performance over its dynamic range and that the lowest detection rates correspond to sweetness (glucose solution), while the highest rates correspond to bitterness (quinine-HCl).

**Figure 10:**
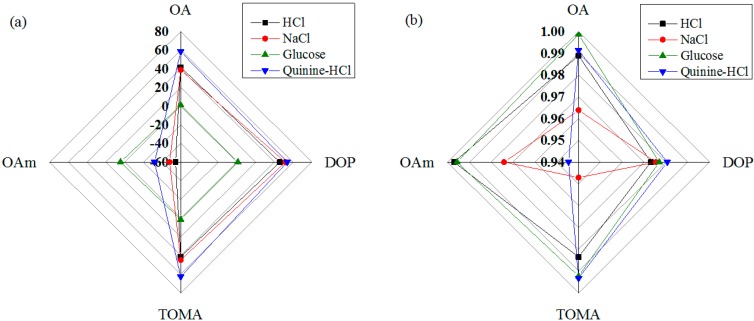
Performance of the proposed sensing system for different taste solutions: (**a**) sensitivity; and (**b**) linearity.

The sensitivities of the four IDC taste sensing elements (S1 to S4) of the array under different taste solutions are shown using a radar chart in Figure 10a. From this chart, it is apparent that the second IDC sensing element, S2, which contains dioctyl phosphate (DOP), has a higher sensitivity than the other elements for most of the taste solutions, for, *i.e.*, saltiness (NaCl) and sourness (HCl). The fourth IDC sensing element, S4, which contains the oleyl amine (OAm)-containing dielectric material, shows the highest response to sweetness (glucose). The sensitivities of the proposed taste sensing system for HCl and glucose are approximately 45.78 mV/decade and 4.39 mV/decade, respectively.

The radar chart in Figure 10b shows the linearity performance of the proposed sensing system under different taste sensing solutions. It is seen that, over the dynamic range, the proposed sensing system has the highest linearity with glucose, with an *R*^2^ value of approximately 0.9958 for OA lipid, and the lowest linearity performance with quinine-HCl, with an *R*^2^ value of approximately 0.94458 for OAm lipid. 

**Figure 11:**
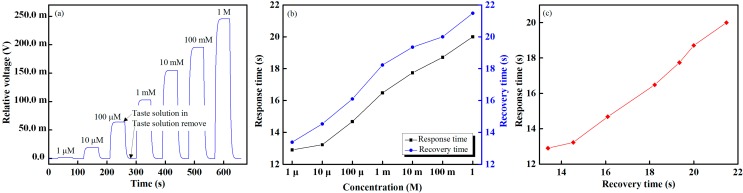
Sensing performance of the IDC taste sensing system: (**a**) real-time taste sensing responses of HCl; (**b**) response and recovery times; and (**c**) response *versus* recovery times at different concentrations of HCl.

**Figure 12:**
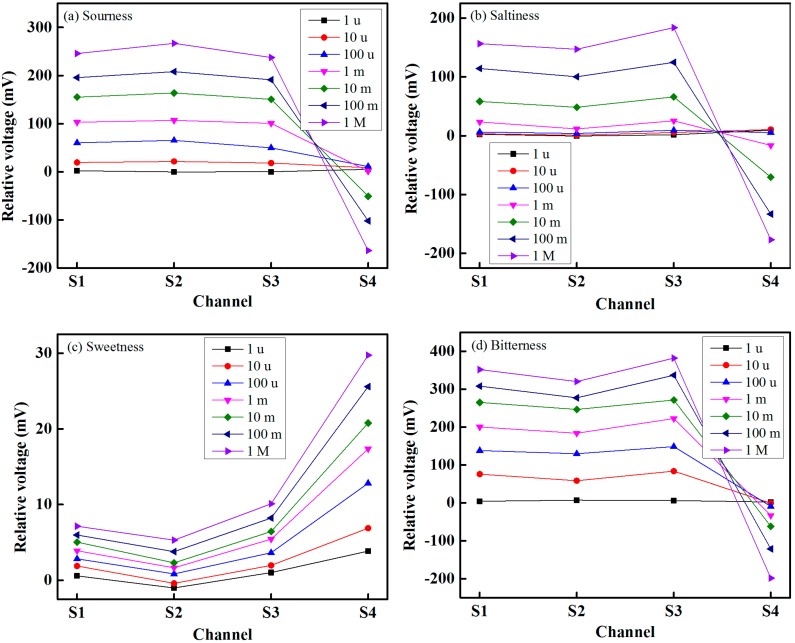
Taste sensing responses of the proposed IDC taste sensing elements of the array under different samples with different levels of concentration: (**a**) sourness; (**b**) saltiness; (**c**) sweetness; and (**d**) bitterness.

The response and recovery times of the proposed IDC taste sensing system are shown in Figure 11. From Figure 11a, it is seen that the proposed IDC taste sensing system has the shortest response and recovery times (12.9 and 13.39 s, respectively). From Figure 11b, it is seen that the response and recovery times are approximately proportional to the increase in the concentration of the taste solution. From Figure 11c, which shows the response *versus* recovery times of HCl at concentrations ranging from 1 µM to 1 M, it is apparent that the response time is proportional to the recovery time. The proposed IDC taste sensing system also offers stable sensing performance over the dynamic range. The responses of the proposed IDC taste sensor array under real samples with different levels of sourness, saltiness, sweetness and bitterness are shown in Figure 12.

To determine the power of discrimination and separation of the proposed IDC taste sensor array, a statistical method, *i.e.*, principle component analysis (PCA), was used for pattern recognition. PCA, which is simple and effective, is the most popular multivariate statistical technique; using orthogonal transformation, PCA converts a group of observations of likely correlated variables into a group of values of linearly-uncorrelated variables (called principal components). PCA presents the pattern of similarities of observations and of variables as points on a graph [42,43]; the direction in the feature space along which the projections have the largest variance is indicated by the first principal component, while the direction that maximizes variance among all directions orthogonal to the first principal component is indicated by the second principal component.

**Figure 13:**
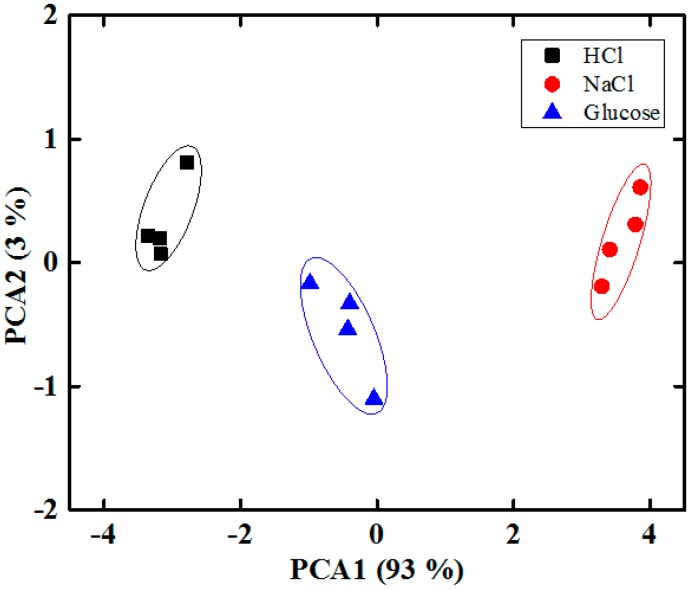
PCA plots of the response to the three different tastes.

**Figure 14:**
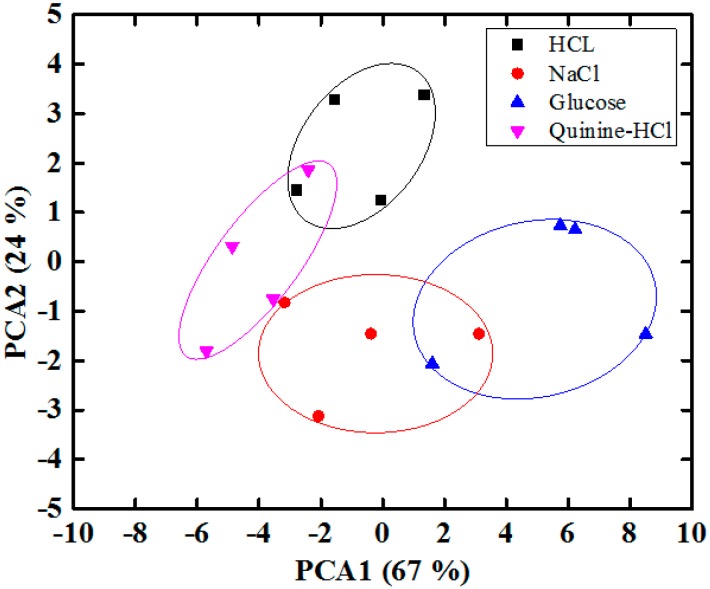
Result of PCA applied to the dataset from the proposed IDC taste sensor array of mixed taste solutions.

We performed a PCA of the data by collecting the maximum relative amplitudes of each sensor over five sensing cycles and converted these into an m × n matrix, where m = 15 is the number of measurements and n = 4 the number of sensors. Figure 13 shows the two-dimensional PCA (PC1 to PC2) score plot for the sensing discrimination of three tastes using the proposed IDC taste sensor array. These results demonstrate that the sensor array successfully distinguishes different types of taste based on the response of the four different sensing elements of the array. We have grouped the three different taste substance into the same ellipse in order to emphasize the separation power of the sensor array. PC1 and PC2 can explain 93% and 3% of the variance, respectively. The total accumulative variance contribution from PC1 and PC2 is therefore 96%, indicating that our proposed IDC taste sensor array is capable of separating different types of taste successfully. To observe the discrimination response of the proposed IDE taste sensor array under the mixed taste substances, we selected several mixture of taste substances, and the PCA plot of the response is shown in Figure 14. From Figure 14, it is found that the proposed IDC sensor array can recognize different types of taste of the mixed complex taste substances. 

We were able to test the performance of our proposed IDC taste sensing system with another taste sensing system that is based on the potentiometry [52] principle, with respect to the dynamic range width, linearity, response time and sensitivity. We found that the proposed sensing system offers a large dynamic range of 1 µM to 1 M (whereas it is 0.1 µM to 0.1 M in [52]) and more linear sensing performance (correlation coefficient of *R*^2^ = 0.9958, approximately), as compared with the potentiometric-taste sensing system. The potentiometric taste [52] sensor gives a stable response after 6.45 min when it is immersed in the taste solution, whereas in the case of the proposed IDC taste sensing system, the sensor array offers a stable response within 12.9 s. Therefore, we can say that the proposed taste sensing system has a short response time. Moreover, the slope, *i.e.*, sensitivity (45.78 mV/decade) of the proposed sensing system, was higher than the potentiometric taste sensor. The commercially available electronic tongue system SA402 [27,53] offers many features compared to our proposed IDC taste sensing system, but we were able to compare the performance of our proposed IDC taste sensing system with the electronic tongue system SA402. The sensitivity of the proposed IDC taste sensor array and the commercially available electronic tongue system SA402 [53,54] for sourness (HCl) was about 45.78 mV/decade and 50 mV/decade, respectively. From this result, it is seen that the electronic tongue system SA402 is more sensitive than the proposed IDC taste sensor array. The response time of the proposed sensor array was about 12.9 s, whereas the response time of the electronic tongue system SA402B [53] was about 20 s, which indicates that the proposed IDC taste sensing system gives a faster response than the electronic tongue system SA402. The overall fabrication process of the proposed IDC sensor array was easier than the SA402 tongue system, but if we consider only the fabrication process of the sensing element, then the fabrication process of the sensing element of the proposed IDC taste sensor array was more complex than the sensing element of the electronic tongue system SA402. Moreover, the fabrication cost of the IDC taste sensor array was about 4000 USD, whereas the fabrication cost of the commercial electronic tongue system SA402 was more expensive than the proposed IDC taste sensor array. 

## 5. Conclusions

In this study, we designed and developed an interdigitated capacitor (IDC) taste sensor array to detect different taste solutions, namely sweetness, saltiness, sourness and bitterness. Four different types of lipid—oleic acid (OA), dioctyl phosphate (DOP), trioctylmethylammonium chloride (TOMA) and oleyl amine (OAm)—were incorporated into polyvinylchloride (PVC), dioctyl phenylphosphonate (DOPP) and tetrahydrofuran (THF) to make the four dielectric materials used in the IDC array sensing elements. The proposed taste sensor array has many advantages, including easy fabrication, high sensitivity, low cost, compactness and linear response over a dynamic range; in addition, its circuitry can be fabricated from readily available and inexpensive electronic components. The highly sensitive taste sensor array was shown to have short response and recovery times of approximately 12.9 and 13.39 s, respectively. According to the experimental results, the dynamic range of the taste sensor array varies from 1 µM to 1 M; the response property is linear, and the reproducibility of the sensing system is high. The most popular multivariate statistical technique, principal component analysis (PCA), was used to determine the power of discrimination and separation of the sensor array. The proposed IDC sensor array successfully classified different types of taste from data collected using its four sensing elements. In future studies, different tastes will be detected, and other sensing materials will be incorporated into the system. We also plan to use the results of this experiment to fabricate an IDC-based gas sensor array.
